# Comparing the Wiltse approach and classical approach of pedicle screw and hook internal fixation system for direct repair of lumbar spondylolysis in young patients: A case-control study

**DOI:** 10.1097/MD.0000000000034813

**Published:** 2023-09-15

**Authors:** Tao Zhang, Lihua Ma, Hua Liu, Chengwei Yang, Songkai Li

**Affiliations:** a Department of Spine Surgery, The 940th Hospital of Joint Logistics Support Force of Chinese PLA, Lanzhou, China; b The First Hospital of Lanzhou University, Lanzhou, China.

**Keywords:** isthmic repair, lumbar spondylolysis, pedicle screw and hook internal fixation system, Wiltse approach

## Abstract

The aim of this study was to investigate the clinical effect of direct isthmus repair via Wiltse approach and classical approach in the treatment of simple lumbar spondylolysis in young patients. Thirty-three patients with simple lumbar spondylolysis underwent direct isthmic repair via the Wiltse approach (n = 17) or the classical approach (n = 16). The operation time, intraoperative blood loss, postoperative drainage volume, hospital stay, fusion rate, visual analogue scale (VAS), and the Oswestry disability index were evaluated and compared between the 2 groups. The amount of intraoperative blood loss, postoperative drainage volume, and the duration of hospital stay in the Wiltse group were lower than those in the classical group (*P* < .05). There was no significant difference in Oswestry disability index score between the Wiltse group and the classical group at 3 months, 6 months, and 1 year after operation, but the visual analogue scale score in the Wiltse group was lower than that in the classical group at 6 months after surgery (*P* < .05). The Wiltse approach was comparable to the classical approach in terms of bone graft fusion time and fusion rate. The Wiltse approach for isthmus repair can achieve the same or even better clinical effect than the classical approach, and the Wiltse approach is more minimally invasive. Pedicle screw-hook internal fixation system combined with autogenous iliac bone graft via Wiltse approach is a feasible, safe, and effective minimally invasive surgical method for the repair of isthmic spondylolysis in young patients.

## 1. Introduction

Lumbar spondylolysis is a bony defect of the pars interarticularis that can occur unilaterally or bilaterally and usually manifests as axial back pain. It is common among young people, especially professional athletes (e.g., weightlifters, gymnasts, etc.) and military personnel.^[1,2]^ Partial patients with early stages of lumbar spondylolysis can obtain satisfactory results through conservative treatment (such as brace use).^[3,4]^ However, isthmic spondylolysis is the pathological basis of lumbar spondylolisthesis. Evidence shows that when chronic, bilateral pars defects develop, 43% to 74% of patients will progress to grade 1 or 2 spondylolisthesis. There is also fair evidence that some patients will develop significant symptoms and will undergo surgical treatment.^[5]^ Timely and effective repair of the isthmus can alleviate the symptoms of lower back pain and effectively prevent the occurrence of lumbar spondylolisthesis. Therefore, when conservative treatment is ineffective, surgery should be performed in patients with lumbar spondylolysis.

At present, the commonly used isthmus defects surgical repair methods include the modified Buck method^[6]^ and the pedicle screw-based method.^[7,8]^ However, Buck technology is less stable than pedicle screw-based methods,^[9]^ thus the pedicle screw-based methods are more prevalent. The classical midline approach requires extensive dissection of the paraspinal muscles, which is easy to lead to scar formation in the operative area, postoperative low back pain, lumbar weakness, and instability. In theory, these problems can be avoided through the Wiltse approach, and it is more in line with the minimally invasive treatment requirements for young patients with lumbar spondylolysis. The Wiltse approach is widely used as a simple and minimally invasive method for thoracolumbar fractures and lumbar degenerative diseases.^[10,11]^ However, no previous reports have compared the Wiltse approach isthmus repair technique with the classical approach. Therefore, this study compared the differences between the Wiltse approach and the classical posterior approach for isthmus repair using a pedicle screw-hook internal fixation system to investigate the advantages of direct repair of isthmus via Wiltse approach in terms of pain relief, clinical effects, and bone graft fusion rate.

## 2. Materials and Methods

### 2.1. Inclusion and exclusion criteria

All cases were screened according to the following criteria. The inclusion criteria were as follows: age ≤ 30 years. All patients had lower back pain, which affected their daily lives or training. The course of the disease was more than 6 months, and there was no obvious relief of lower back pain after standard conservative treatment. Imaging confirmed single-segment bilateral isthmic spondylolysis without prolapse of the lumbar intervertebral disc, lumbar spondylolisthesis, rhachischisis, etc. Magnetic resonance imaging of the lumbar spine assessed intervertebral disc degeneration as grade I or grade II.^[12]^ The cause of the low back pain was confirmed to be the isthmic cleft. Volunteer for surgery and participate in this study. The exclusion criteria included: single-segment unilateral isthmic spondylolysis, multi-segment isthmic spondylolysis, isthmic spondylolysis with intervertebral disc herniation, lumbar spondylolisthesis, etc, or accompanied by root radiation pain symptoms in the lower extremities. The source of lower back pain could not be determined. Patients cannot cooperate with postoperative treatment. History of lumber surgery. Inoperable due to other diseases.

### 2.2. General materials

A total of 33 patients with lumbar spondylolysis, according to the inclusion and exclusion criteria, were recruited and admitted from January 2016 to November 2019. All patients had significant symptoms of lower back pain, which worsened when standing for long periods or bending and overloading, or when stretching the back. Seven patients had unilateral buttock pain and discomfort. Physical examination showed that the sensation, muscle strength, reflex, and pathological signs of the lower limbs were negative in all patients; mild tenderness near the paraspinous process of lumbosacral region; and lumbar flexion and extension activity was slightly limited. All patients underwent lumbar anteroposterior, lateral, extension and flexion X-ray. And lumbar 3-dimensional computer tomography (CT) and magnetic resonance imaging were performed at the same time. The patients were divided into 2 groups by using random number tables: the Wiltse group was treated using the Wiltse approach (n = 17), and the classical group was treated using the classical posterior midline approach (n = 16). All surgeries were performed by the same surgeon.

### 2.3. Ethical compliance

This study was approved by the Institutional Ethical Board of the 940th Hospital of Joint Logistics Support force of Chinese PLA. The study approval number was 2016KYLL002.

### 2.4. Surgical procedure

#### 2.4.1. Wiltse approach^[13]^.

**Figure 1.**
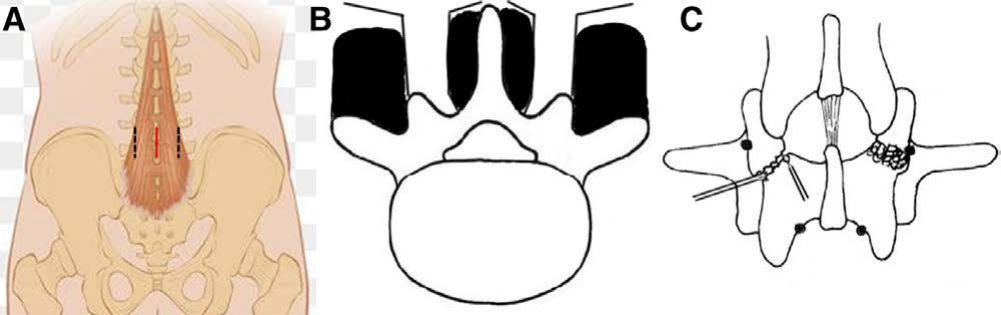
Schematic representation of the surgical method of the Wiltse approach. (A) The red line in the middle shows a skin incision. Bilateral black dotted lines show fascia incisions. (B) Expose the interspace between multifidus muscles and longissimus muscles. (C) Treatment of scaring and iliac bone grafting.

After general anesthesia, the patients were placed in the prone position. Located the injured segments by “C”-arm X-ray and marked it on the body surface. A 5 cm longitudinal incision was made in the posterior midline of the lower back (Fig. 1A). Subcutaneous tissue was separated from the surface of the lumbodorsal fascia to 2 cm from the midline. The bilateral fascial layers were cut along the interspace between the multifidus and longissimus. The outer edge of the articular process, the isthmus, and part of the lamina were exposed. Capsuloligamentous structures of the adjacent facet joint should be preserved. A minimally invasive retractor was used (Fig. 1B). Weinstein’s slight lateral positioning method was used to place the pedicle screws to reduce the influence of bilateral facet joints. The scar and hyperplastic tissue between the stumps of the isthmus were completely removed. The bone graft bed was prepared by grinding the sclerotic bone at the broken end of the isthmus with a drill bit (Fig. 1C). Iliac bone blocks and cancellous bone particles were harvested from the posterior superior iliac spine under the same skin incision. The appropriately trimmed iliac bone block was knocked and implanted into the isthmic defect (Fig. 1C). Lamina hooks were placed at the lower edge of the affected lamina. After pre-bending, a connecting rod of an appropriate length was selected to connect the pedicle screw to the lamina hook. Moderate pressure was applied to the isthmus stump and the stability of the bone graft was checked. Residual cancellous bone particles were implanted around the isthmus. A drainage tube was then placed and sutured layer by layer.

#### 2.4.2. Classical approach.

The method of anesthesia and positioning were the same as for the Wiltse approach. A 6-cm longitudinal incision was made at the posterior midline of the lower back. The skin, subcutaneous tissue, and deep fascia were dissected sequentially. Bilateral sacrospinous muscles were dissected from the periosteum of the spinous process and lamina to expose the facet joint and lamina of the diseased segment. The single-tooth retractor fully exposed the bilateral lamina, isthmus, and facet joint, and the rest of the operation was the same as the Wiltse approach.

### 2.5. Postoperative observation and follow-up

The drainage tube was removed when the drainage volume was less than 50 mL/24 h after operation. Antibiotics were routinely used within 24 hours after surgery. Patients can migrate under the protection of the brace 3 days after operation and wear it for at least 3 months. Lumbar X-ray film was reexamined at 2 to 3 days after operation, and lumbar 3-dimensional CT was reviewed every 3 months to evaluate the bone healing and healing time of isthmus. Bone graft fusion criteria: 3-dimensional CT of lumbar spine was used to evaluate the continuous bone trabeculae at the isthmus stump. Visual analogue scale (VAS) score and Oswestry disability index (ODI) were used to observe the improvement of low back pain at 3 months, 6 months, and 1 year after operation.

### 2.6. Data analysis

All data were analyzed by SPSS20.0 software (IBM Corp., Armonk, NY), and the measurement data were expressed as mean ± standard deviation. The differences were compared using *t* test or chi-square test, and the statistical significance level was defined as *P* < .05.

## 3. Results

Overall, there were 17 patients (16 males, 1 female) in the Wiltse group, and 16 patients (16 males) in the classical group. All isthmus injury segments were located at L4 and L5, of which L5 was the most common segment (84.8%, 28/33). The average age of patients in the Wiltse group and the classical group was (22.2 ± 2.2) years (range, 19–27 years) and (22.8 ± 2.2 years) (range, 19–28 years), and the mean course of the disease was (14.6 ± 3.7) months and (14.2 ± 4.0) months, respectively. There were no significant between-group differences regarding the location of the isthmus segment, age, and disease course (Table 1).

**Table 1:** Baseline characteristics.

Parameter	The Wiltse group	The classical group	*P*
Patients (female/male)	17 (1/16)	16 (0/16)	–
Mean age in year	22.2 ± 2.2	22.8 ± 2.2	.465
Average course of disease (mo)	14.6 ± 3.7	14.2 ± 4.0	.735
Location of spondylolysis			
L4	3	2	1.000
L5	14	14	
Operation time (min)	108.2 ± 16.7	102.5 ± 16.5	.329
Blood loss (mL)	95.9 ± 26.5	121.9 ± 21.7	.004
Postoperative drainage volume (mL)	23.2 ± 6.8	63.8 ± 12.0	<.001
Duration of hospitalization (d)	8.3 ± 1.0	11.5 ± 1.5	<.001
Follow-up in months (mo)	18.7 ± 6.8	18.8 ± 4.7	.959
Fusion time (m)	7.6 ± 2.4	6.9 ± 1.8	.388
One year postoperative fusion rate	82.4% (14/17)	93.8% (15/16)	.601

The operation time was (108.2 ± 16.7) minutes in the Wiltse group (range, 90–150 minutes) and (102.5 ± 16.5) minutes (range, 80–140 minutes) in classical groups (*P* = .329). The intraoperative blood loss was (95.9 ± 26.5) mL (range, 50–150 mL) in the Wiltse group and (121.9 ± 21.7) mL (range, 100–150 mL) in the classical group (*P* = .004). The average postoperative drainage volume was (23.2 ± 6.8) mL (range, 10–30 mL) in the Wiltse group and (63.8 ± 12.0) mL (range, 50–80 mL) in the classical group (*P* < .001). The hospital stay was (8.3 ± 1.0) days (range, 7–10 days) in the Wiltse group and (11.5 ± 1.5) days (range, 10–15 days) in the classical group (*P* < .001). The intraoperative blood loss, postoperative drainage volume, and hospital stay in the Wiltse group were significantly lower than those in the classical group, and the differences were statistically significant (Table 1).

The preoperative VAS and ODI scores of the Wiltse group and classical group were (5.9 ± 0.8, 41.8% ± 7.5%) and (5.6 ± 0.7, 40.5% ± 3.2%), respectively. The preoperative VAS and ODI scores in the Wiltse group were similar to those in the classical group (*P* = .225, *P* = .541, Table 2). The symptoms of lower back pain were significantly relieved after operation. In the Wiltse group, the VAS and ODI scores decreased from (5.9 ± 0.8) and (41.8% ± 7.5%) to (1.2 ± 0.8) and (11.8% ± 3.5%) at 3 months after surgery. In the classical group, the VAS and ODI scores decreased from (5.6 ± 0.7) and (40.5% ± 3.2%) to (1.6 ± 0.6) and (13.5% ± 4.1%) at 3 months after surgery. There was no significant difference in VAS and ODI scores between the 2 groups at 3 months after operation (*P* = .116, *P* = .201, Table 2). Compared with 3 months after operation, VAS and ODI scores were further improved at 6 months and 1 year after operation. At 6-month follow-up, the VAS score of the Wiltse group was (0.8 ± 0.6), which was significantly lower than that of the classical group (1.4 ± 0.5) (*P* = .010, Table 2), but the ODI scores between the 2 groups were similar (*P* = .161). There was no difference in VAS and ODI scores between the 2 groups at 1-year after operation (*P* = .886, *P* = .571, Table 2).

**Table 2:** The VAS score and ODI for low back pain at different time between groups.

	VAS			ODI		
	The Wiltse group	The classical group	*P*	The Wiltse group	The Classical group	*P*
Preoperation	5.9 ± 0.8	5.6 ± 0.7	.225	41.8% ± 7.5%	40.5% ± 3.2%	.541
Postoperation 3 mo	1.2 ± 0.8	1.6 ± 0.6	.116	11.8% ± 3.5%	13.5% ± 4.1%	.201
Postoperation 6 mo	0.8 ± 0.6	1.4 ± 0.5	.010	10.9% ± 3.2%	12.6% ± 3.6%	.161
Postoperation 1 yr	0.4 ± 0.5	0.4 ± 0.5	.886	9.8% ± 2.9%	9.3% ± 2.2%	.571

ODI = Oswestry disability index, VAS = visual analogue scale.

**Figure 2.**
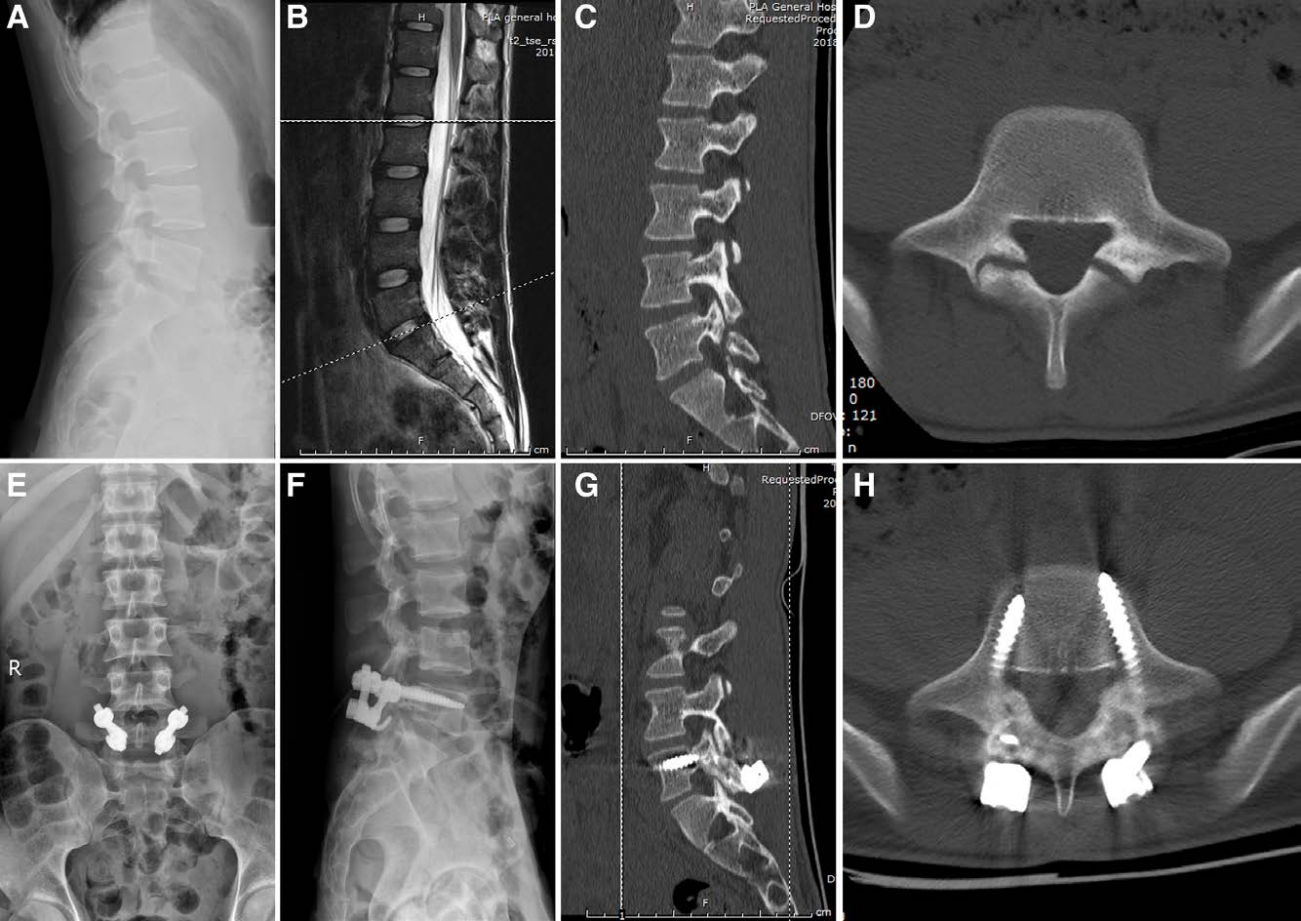
A 19-year-old man with a symptom of lower back pain for 1 year. (A) Preoperative lateral X-ray of the lumbar spine shows L5 spondylolysis. (B) Preoperative sagittal MRI of the lumbar spine shows no significant disc degeneration. (C and D) Preoperative sagittal and axial CT shows bilateral pars defect in the L5 isthmus. (E and F) Three days after the operation, the X-ray film shows a good internal fixation position. (G and H) Sagittal and axial CT shows bilateral isthmus bony healing at 1 year after surgery. CT = computer tomography, MRI = magnetic resonance imaging.

The mean follow-up time was (18.7 ± 6.8) months (range, 12–36 months) in the Wiltse group and (18.8 ± 4.7) months (range, 12–28 months) in the classical group. There was no significant difference in the average follow-up time between the 2 groups (*P* = .959, Table 1). The average fusion time of the bone grafts was (7.6 ± 2.4) months in the Wiltse group and (6.9 ± 1.8) months in the classical group (*P* = .388, Table 1). At 1 year after surgery, 3 cases of isthmic bone graft did not fused in the Wiltse group and 1 case in the classical group. Figure 2 shows a typical case of lumbar spondylolysis with screw-hook internal fixation and bone graft fusion surgery using the Wiltse approach.

No nerve root injury or dural tear occurred during the surgery. All skin incisions healed in one stage after surgery, no hematoma was observed at the site of iliac crest extraction. During the follow-up, no secondary adjacent segment degeneration, loosening, or breakage of internal fixation in either group.

## 4. Discussion

Lower back pain due to spondylolysis is caused by microtraumatic overloading and overuse of the isthmus before developing spondylolisthesis and due to discopathy.^[14,15]^ As a matter of fact, failing the correct functioning of the isthmus, discopathy will be accelerated as the disc will be the only guarantor of segmental spinal stability, and consequently, it will be subject to overload. In addition, if left untreated in moderately active patients, bone healing is as low as 0% for bilateral spondylolysis.^[5,14]^ There is also fair evidence that some patients with early-stage spondylolysis will develop significant symptoms and undergo segmental fusion treatment as adults.^[5]^ For some patients with early lumbar spondylolysis, the symptoms of lower back pain can be treated with conservative methods, such as immobilization using a brace, bed rest, and avoiding bending of the waist and overloading.^[4,16]^ Thus, all patients with lumbar spondylolysis in our department need to undergo normative conservative treatment with a brace and rest, so the average course of disease is more than 1 year. However, for most patients who underwent failed conservative treatment, lumbago symptoms usually interfere with daily life and work tasks. Therefore, early surgical treatment is necessary for patients who fail in conservative treatment. Surgical repair of pars defect can enable young patients with spondylolysis to return to work as soon as possible.^[3,17]^

At present, the commonly used surgical methods for repair isthmus defects include modified Buck method,^[6,18]^ screw-rod technique,^[19,20]^ screw-hook fixation technology,^[7,21]^ and screw-rod-screw short-segment fixation with direct repair of isthmus.^[8,22]^ Screw-rod-screw short-segment fixation affects the spinal motor unit and limits local movement, especially in the lumbosacral region. Short-segment applications are rare. Other internal fixation techniques are single-segment fixation, which preserves normal spinal motor units, and are widely used, with less damage to spinal anatomy. The screw-hook internal fixation system has better biomechanical properties than the screw-rod and Buck’s internal fixation system.^[7,9]^ Moreover, patients with screw-hook fixation are more likely to return to work earlier than those using the Buck method.^[21]^ Therefore, we prefer to repair the isthmus by screw-hook internal fixation to allow young patients to return to work faster and better.

Wiltse technique has become an increasingly popular minimally invasive approach in thoracolumbar surgery,^[11,23]^ and has been reported to have the advantages of shorter operation time and hospital stay, less bleeding, lower cost, and lower incidence of complications.^[24]^ In this study, intraoperative blood loss, postoperative drainage volume, and hospital stay of the Wiltse approach were significantly lower than those of classical group, indicating that the Wiltse technique is a relatively less invasive isthmus repair approach, which is consistent with other reports.^[25,26]^ However, in this study, the operation time of the Wiltse group was slightly longer than that of the classical group, which was inconsistent with the report,^[24]^ but there was no significant difference in the operation time between the 2 groups. This is mainly due to the smaller space available for the intramuscular approach, which makes it more difficult to place the hook and connect the screw-rod-hook complex. Therefore, a suitable retractor is essential for isthmus repair via the Wiltse approach.

Besides, in this study, ODI scores at 3 months, 6 months, and 1 year after surgery were significantly lower than preoperative values, but there was no significant difference between the Wiltse approach and the classical approach. At the same time, there was no difference in VAS score between the Wiltse approach and the classical approach at 3 months and 1 year after surgery. However, the VAS score of the Wiltse approach was significantly lower than that of the classical approach at 6 months after operation. Compared with the classical approach, the Wiltse approach can achieve better clinical outcomes in repairing the isthmus and is less invasive, which is more in line with the minimally invasive treatment needs of patients. Therefore, isthmus repair via Wiltse approach is a feasible and minimally invasive method for lumbar spondylolysis.

The reasons for the satisfactory clinical results of the Wiltse approach for isthmus repair are as follows: the Wiltse approach can significantly reduce damage to the multifidus muscle^[27]^ without dissection of the muscle, providing direct access and exposing the isthmus and facet joints. In the intermuscular approach, the sacrospinalis muscle does not need to be overstretched to avoid ischemic necrosis of the muscle.^[27]^ In addition, the screw-hook internal fixation system is a single-segmental fixation method that does not damage the normal spinal motor unit and approaches the normal physiological status.

The following points should be paid attention to in the repair of lumbar spondylolysis by pedicle screw-hook internal fixation combined with autogenous iliac bone grafting through Wiltse approach: to avoid iatrogenic capsular injury, pedicle screws should be placed above the root of the transverse process, away from the isthmus and articular process. Keeping the pedicle screw away from the isthmus facilitates clearance of the scar tissue in the isthmus and local bone grafting. When dissecting to expose the lower margin of the lamina, it should be tilted inward to avoid exposure of the inferior articular process. Excessive exposure of the outer edge of the lamina will damage the inferior articular process. A cancellous bone block of appropriate size was selected to knock in between the broken ends of the isthmus, and cancellous bone particles were implanted around the isthmus to ensure local bone graft fusion. To avoid shortening of the isthmus and interfering with the occlusion relationship of the inferior and superior articular process, moderate pressure was applied to make the isthmus stump close to the bone graft when tightening the pedicle screw and lamina hook. Excessive compression between screws and hooks may cause facet joint degeneration and lower back pain. All these surgical details determine a better clinical outcome.

Both the Wiltse group and the classical group were followed up for more than 12 months. There was no significant difference in the postoperative bone graft fusion time between the 2 groups, but the average bone graft fusion time in the Wiltse group (7.6 ± 2.4 months) was slightly longer than that in the classical group (6.9 ± 1.8 months). However, the mean time of bone graft fusion in both groups was significantly lower than 13 months reported in the literature.^[22]^ After a follow-up of more than 1 year, the fusion rate of isthmus in the Wiltse group (82.4%) was not significantly different from that in the classical group (93.8%). The fusion rate of isthmus in both groups was higher than that reported in the literature (77.7%).^[28]^ In terms of fusion time and bone graft fusion rate, the Wiltse approach can achieve the same effect as the classical approach. Satisfactory bone graft fusion was obtained in all cases, which may be related to strict case selection and good isthmus management. All the patients were relatively young, the sclerotic bone in the isthmus stump was completely removed during the operation, the bone graft in the isthmus was reliable, and the patients’ compliance was good.

## 5. Conclusions

The Wiltse approach for isthmus repair can achieve the same or even better clinical effect than the classical approach, and the Wiltse approach is more minimally invasive. Pedicle screw-hook internal fixation system combined with autogenous iliac bone graft via Wiltse approach is a feasible, safe, and effective minimally invasive surgical method for the repair of isthmic spondylolysis in young patients, and short-term follow-up results are good.

## Author contributions

**Conceptualization:** Tao Zhang.

**Data curation:** Tao Zhang, Hua Liu.

**Formal analysis:** Lihua Ma, Chengwei Yang.

**Funding acquisition:** Songkai Li.

**Investigation:** Tao Zhang, Hua Liu, Chengwei Yang.

**Methodology:** Lihua Ma.

**Project administration:** Songkai Li.

**Resources:** Chengwei Yang.

**Software:** Hua Liu.

**Supervision:** Songkai Li.

**Writing – original draft:** Tao Zhang, Lihua Ma.

**Writing – review & editing:** Songkai Li.
